# A Network of Conserved Damage Survival Pathways Revealed by a Genomic RNAi Screen

**DOI:** 10.1371/journal.pgen.1000527

**Published:** 2009-06-19

**Authors:** Dashnamoorthy Ravi, Amy M. Wiles, Selvaraj Bhavani, Jianhua Ruan, Philip Leder, Alexander J. R. Bishop

**Affiliations:** 1 Greehey Children's Cancer Research Institute, University of Texas Health Science Center at San Antonio, San Antonio, Texas, United States of America; 2 Department of Computer Science, University of Texas at San Antonio, San Antonio, Texas, United States of America; 3 Harvard Medical School, Department of Genetics, Harvard University, Boston, Massachusetts, United States of America; University of California San Diego, United States of America

## Abstract

Damage initiates a pleiotropic cellular response aimed at cellular survival when appropriate. To identify genes required for damage survival, we used a cell-based RNAi screen against the *Drosophila* genome and the alkylating agent methyl methanesulphonate (MMS). Similar studies performed in other model organisms report that damage response may involve pleiotropic cellular processes other than the central DNA repair components, yet an intuitive systems level view of the cellular components required for damage survival, their interrelationship, and contextual importance has been lacking. Further, by comparing data from different model organisms, identification of conserved and presumably core survival components should be forthcoming. We identified 307 genes, representing 13 signaling, metabolic, or enzymatic pathways, affecting cellular survival of MMS–induced damage. As expected, the majority of these pathways are involved in DNA repair; however, several pathways with more diverse biological functions were also identified, including the TOR pathway, transcription, translation, proteasome, glutathione synthesis, ATP synthesis, and Notch signaling, and these were equally important in damage survival. Comparison with genomic screen data from *Saccharomyces cerevisiae* revealed no overlap enrichment of individual genes between the species, but a conservation of the pathways. To demonstrate the functional conservation of pathways, five were tested in *Drosophila* and mouse cells, with each pathway responding to alkylation damage in both species. Using the protein interactome, a significant level of connectivity was observed between *Drosophila* MMS survival proteins, suggesting a higher order relationship. This connectivity was dramatically improved by incorporating the components of the 13 identified pathways within the network. Grouping proteins into “pathway nodes” qualitatively improved the interactome organization, revealing a highly organized “MMS survival network.” We conclude that identification of pathways can facilitate comparative biology analysis when direct gene/orthologue comparisons fail. A biologically intuitive, highly interconnected MMS survival network was revealed after we incorporated pathway data in our interactome analysis.

## Author Summary

Cellular damage is known to elicit a pleiotropic response, but the relative importance of the constituent components in cell survival is poorly understood. To provide an unbiased identification of the proteins utilized in damage survival, we performed an RNAi survival screen in fly cells with methyl methanesulfonate (MMS). The genes identified are involved in 13 biologically diverse pathways. Comparison with analogous yeast data demonstrated a lack of conservation of the individual MMS survival genes but a conservation of pathways. We went on to demonstrate the MMS responsiveness for five representative pathways in both fly and mouse cells. We conclude that identification of pathways can facilitate comparative biology analysis when direct gene/orthologue comparisons fail. Incorporation of pathway data in interactome analysis also improved connectivity and, more importantly, revealed a biologically intuitive, highly inter-connected “MMS survival network.” This pathway conservation and inter-connectivity implies extensive interaction between pathways; for diseases such as cancer, such crosstalk may dictate disparate cellular responses not necessarily expected and confound treatments that are not tailored to the individual molecular context.

## Introduction

Cellular damage is a normal component of life, with constant damage exposure from both endogenous and exogenous sources. Damage to DNA is considered to be the most biologically relevant lesion with the potential of mutagenic results, though most exogenous agents have the potential to damage many components of the cell. Responding appropriately to such insults, either mitigating cellular toxicity or initiating an appropriate cell death response, is critical, particularly in multi-cellular organisms. Inappropriate responses may facilitate deleterious effects, such as a destabilized genome and diseases such as cancer [1]. As such, DNA damage response (DDR) components are critical suppressors of deleterious effects of genotoxic agents by controlling cell cycle progression, DNA repair, and apoptosis [2]. For this reason, there have been many investigations using a variety of model organisms to identify components of DDR and subsequently to determine how they function and the consequences of their dysfunction. Recent reports suggest that DDR may involve pleiotropic cellular processes other than the central DDR components [3],[4], yet an intuitive systems level view of the cellular components required for damage survival, their interrelationship, and their contextual importance has been lacking. The most comprehensive attempt at understanding the interrelationship of damage response and survival components in yeast at a systems level has been mapping identified genes to a network by integrating the general biological processes as distinct modules [5]. It has been suggested that the inclusion of well-defined biological pathways in protein networks might provide a better understanding of biological interactions therein [6],[7].

In order to identify genes involved in damage response in an unbiased manner and to put them in a functional context, we used an RNAi library [8] to knock-down every predicted protein in the *Drosophila melanogaster* genome and assessed whether knock-down of individual proteins significantly altered cell viability following methyl methanesulfonate (MMS) exposure. MMS is a prototypical alkylating agent that attacks nucleophilic groups, such as those found in nucleic acid [9]. The resultant base methylation destabilizes the glycosidic bond, facilitating the production of the most biologically relevant cellular lesion of MMS, an abasic site in DNA. Other common sources of alkylation damage include endogenous S-adenoysyl methionine [10], the tobacco carcinogen N-nitorsoamine [11] and chemotherapeutics such as temozolomide [12], carmustine (BCNU) [9], and cyclophosphamide [13]. Considering the physiological relevance of alkylation damage, several recent studies using yeast as model organism investigated the global effects of alkylation damage induced by MMS. Viability [14], gene transcription [15] and protein expression [16] were measured in these studies, all of which provided insights into the diverse nature of biological responses to alkylation damage. However when transcriptional responses to various environmental stresses were compared between mammalian and yeast cells [17], Murray *et al.* reported clear distinctions and suggested that this might be the result of different selective pressures between multi-cellular organisms and single-cell organisms. In the attempt to understand diverse responses required for damage survival in a system that is evolutionarily closer to mammals than yeast, we performed a genome-wide, RNAi-based screen using cells derived from *Drosophila melanogaster*. Our experiments were based on “loss of function” and an assessment for cellular viability following exposure to MMS. Here we present results from this MMS survival screen, the pathways that were identified, and a comparative analysis of pathways to understand conservation of these pathways in yeast and mouse cells. Additionally, we present a novel approach of assembling a protein interaction network based on defined biological pathways, which facilitates network consolidation. By including biological pathways in our genomic data and protein network analysis, we were able to determine the commonality across species, which was obscured by direct orthologue comparison, and provide a simplified representation of the global survival responses that we identified.

## Results

### Genome-wide RNAi screen and validation

**Figure 1:**
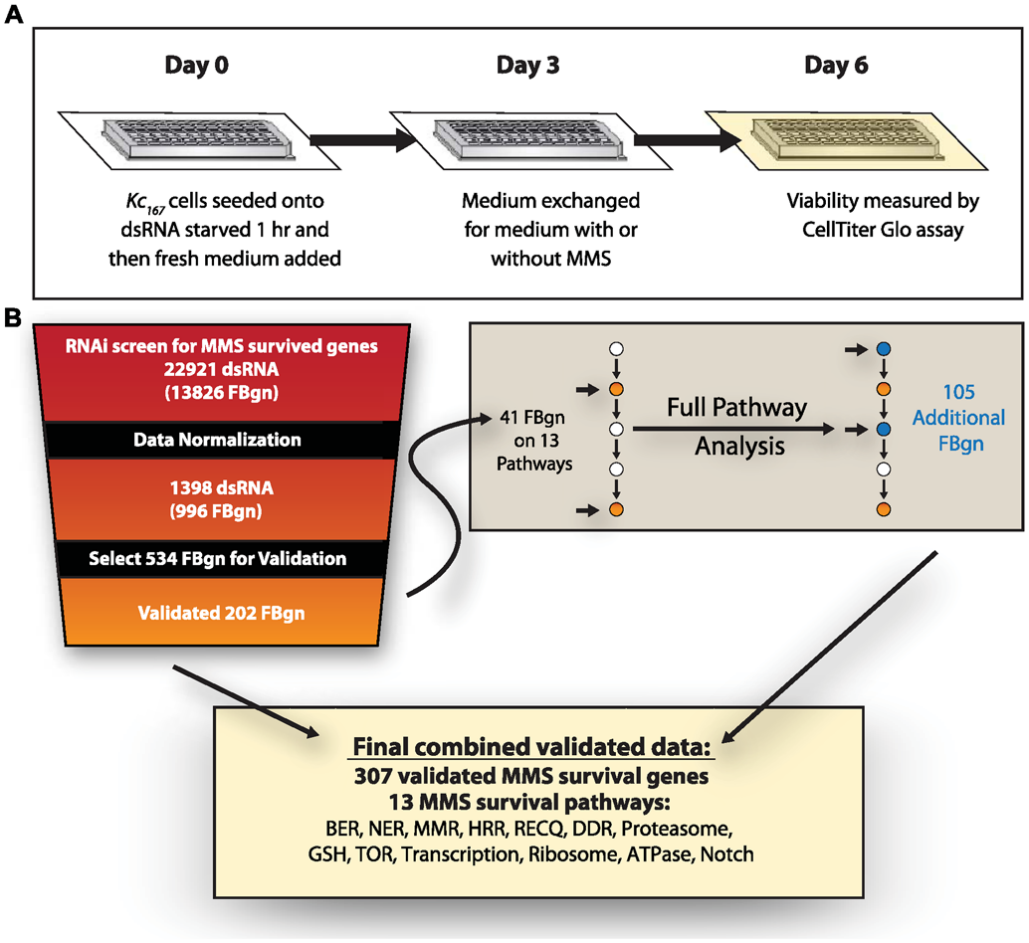
The experimental design of the *Drosophila* RNAi MMS survival screen and summary of the workflow and results. (A) A schematic representation of the experimental design used to screen for genes involved in survival of an MMS exposure using RNAi in *Drosophila*. (B) A schematic representation of the workflow from the initial RNAi screen, the normalization and validation of results, and the follow-up work conducted, with a summary of the number of dsRNA amplicons and genes (FBgn; FlyBase gene number) involved at each step in the process. Also provided are the final numbers of MMS survival proteins and MMS survival pathways (BER: base excision repair; NER: nucleotide excision repair; MMR: mismatch repair; HRR: homologous recombination repair; RECQ: RecQ helicases; DDR: DNA damage response; Proteasome; GSH: glutathione synthesis; TOR: TOR pathway; Transcription: basal transcription; Ribosome; ATPase; Notch: Notch signaling pathway).

An RNAi screen was designed to identify those genes that modulate cellular survival following exposure to a level of MMS-induced damage that resulted in only a limited amount of cell death. A linear decrease in cell viability was observed from 0.002% (w/v) of MMS to 0.008% (w/v) of MMS (Figure S1). A dose of 0.004% (w/v; 40 µg/mL) MMS was chosen, which resulted in a statistically significant decrease to approximately 65% viability, while allowing an additional, measurable decrease in cell survival. RNAi screens were performed using the *Drosophila* RNAi Screening Center (DRSC) version 1 library (about 23,000 *D. melanogaster* open reading frames) [8]. *Kc_167_ Drosophila* cells were exposed to three days of dsRNA to allow protein knock-down, followed by three days of growth in either the absence or presence of MMS exposure (Figure 1A).

To identify those genes required for cell viability following MMS treatment, viability results from the MMS treated RNAi screen were compared with that of the untreated (control) screen as previously described [18]. 1,398 different open reading frames were identified that affected MMS survival in a continuous distribution of cell survival values (see [18] and http://gccri.uthscsa.edu/ABPublished_Data.asp for original data), of which 996 had a unique assigned FlyBase gene number (FBgn; denotes known genes) (see Figure 1B and Table 1). Of these 996 genes, the top 537 were selected for validation analysis by a previously described, stringent validation method [18]. Whereas in [18] we validated normalization methods, here we independently validated individual genes using dsRNA targeting a different region of each gene. 202 protein knock-downs validated with a significant MMS viability effect, while 55 more had a notable trend effect without meeting our stringent statistical criteria (Table S1 and Table S2). An examination of gene ontology (GO) on the 202 validated MMS survival genes revealed a significant enrichment for genes involved in DNA metabolism, gene transcription, and cellular metabolism (Table 2). The overall variety of GO categories observed was quite broad, consistent with the findings reported for an analogous yeast screen [3] (data not shown). Surprisingly though, no significant enrichment between the gene orthologues for the two organisms was observed (G-test p-values of 0.29 and 0.057 or Fisher Exact test p-values of 0.37 and 0.08, yeast and fly, respectively). To further test this, we examined the MMS sensitivity following knock-down of 183 fly genes that were orthologues of to 118 yeast MMS survival genes [3], but only found conservation of MMS survival phenotype with 20 (Table S3). This apparent lack of gene enrichment between species has also been reported when comparing transcriptional profiles between mammalian and yeast cells in response to a variety of stresses [17]. Overall, these results suggest that there may be conservation of the biological processes that respond to MMS rather than the individual genes.

**Table 1:** Summary of MMS survival genes identified by genome-wide RNAi screen.

Normalized RNAi screen results			
	Death	Resistance	Total
**BS_QN**	1183	40	**1223**
**cellHTS2**	594	40	**634**
**total**	1326	72	**1398**
**FBgn**	935	61	**996**

Numbers of death and resistance hits found by background subtraction followed by quantile normalization (BSQN) and cellHTS2 are given. The total number of unique hits and the total number of hits whose amplicons map to genes with FlyBase gene number (FBgn) are also given.

**Table 2:** Categories of Gene Ontology enrichment within the validated MMS survival genes.

Gene Ontology enrichment	
Biological Process	
Adjusted P-value	GO Attribute
<0.001	0006139: nucleobase, nucleoside, nucleotide and nucleic acid metabolic process
<0.001	0006351: transcription, DNA-dependent
<0.001	0032774: RNA biosynthetic process
<0.001	0016070: RNA metabolic process
<0.001	0045449: regulation of transcription
<0.001	0065004: protein-DNA complex assembly
<0.001	0016043: cellular component organization and biogenesis
<0.001	0051252: regulation of RNA metabolic process
<0.001	0006367: transcription initiation from RNA polymerase II promoter
<0.001	0006355: regulation of transcription, DNA-dependent
<0.001	0065003: macromolecular complex assembly
<0.001	0008283: cell proliferation
0.001	0035080: heat shock-mediated polytene chromosome puffing
0.014	0006911: phagocytosis, engulfment
0.018	0045165: cell fate commitment
0.034	0030154: cell differentiation
0.049	0006259: DNA metabolic process

Molecular Function	
Adjusted P-value	GO Attribute
<0.001	0030528: transcription regulator activity
<0.001	0003676: nucleic acid binding
<0.001	0016251: general RNA polymerase II transcription factor activity
<0.001	0003702: RNA polymerase II transcription factor activity
0.003	0005515: protein binding
0.003	0043565: sequence-specific DNA binding

Cellular Component	
Adjusted P-value	GO Attribute
<0.001	0005634: nucleus
<0.001	0005654: nucleoplasm
<0.001	0043227: membrane-bounded organelle
<0.001	0031981: nuclear lumen
<0.001	0016591: DNA-directed RNA polymerase II, holoenzyme
<0.001	0031974: membrane-enclosed lumen
<0.001	0043234: protein complex
<0.001	0032991: macromolecular complex
<0.001	0005667: transcription factor complex
0.003	0000119: mediator complex
0.012	0008540: proteasome regulatory particle, base subcomplex
0.015	0005665: DNA-directed RNA polymerase II, core complex
0.032	0005675: holo TFIIH complex
0.038	0000123: histone acetyltransferase complex

An examination of the 202 validated screen MMS survival genes for category enrichment within Gene Ontologies; Biological Process, with 46 genes having no functional annotation; Molecular Function, with 40 genes having no functional annotation; Cellular Component, with 68 genes having no cellular component annotation, using FuncAssociate.

### Identification of pathways responsive to MMS

**Figure 2:**
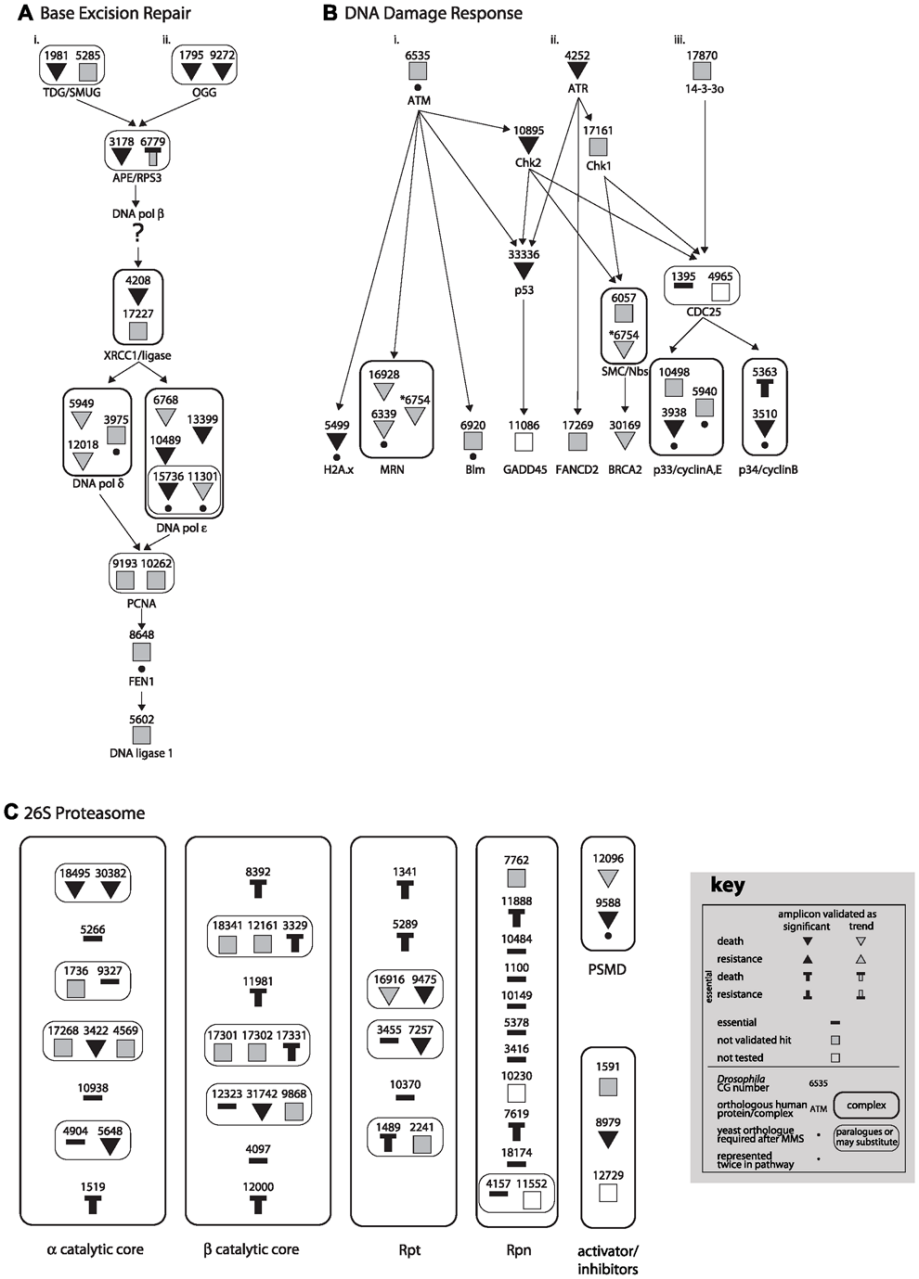
Base excision repair, DNA damage response, and proteasome pathways utilized for MMS survival. CG numbers are given for each *Drosophila* pathway component, as well as the protein names or complex names for their human orthologues. Pathway entry points are noted with Roman numerals at the top, and end points are at the bottom. A key for the following symbols is provided. Symbols encircled with thick lines represent proteins that act together or in a complex, while symbols encircled with thin lines represent paralogues or proteins that may substitute for one another. Proteins found to affect MMS survival are noted as down (death) or up triangles (resistance). Statistically significant proteins are indicated with black triangles, while trend hits are indicated with grey triangles. Essential genes are noted with a thick bar and any with downwards or upwards pointing boxes were also validated as conferring death or resistance, respectively, to MMS upon knock-down. Shaded squares are proteins not found to be hits after validation, and open squares were not tested in our validation. Yeast orthologues previously found to be required for MMS survival [3] are noted with a dot under the symbol. (A) Base Excision Repair. (B) DNA Damage Response. (C) Proteasome.

**Figure 3:**
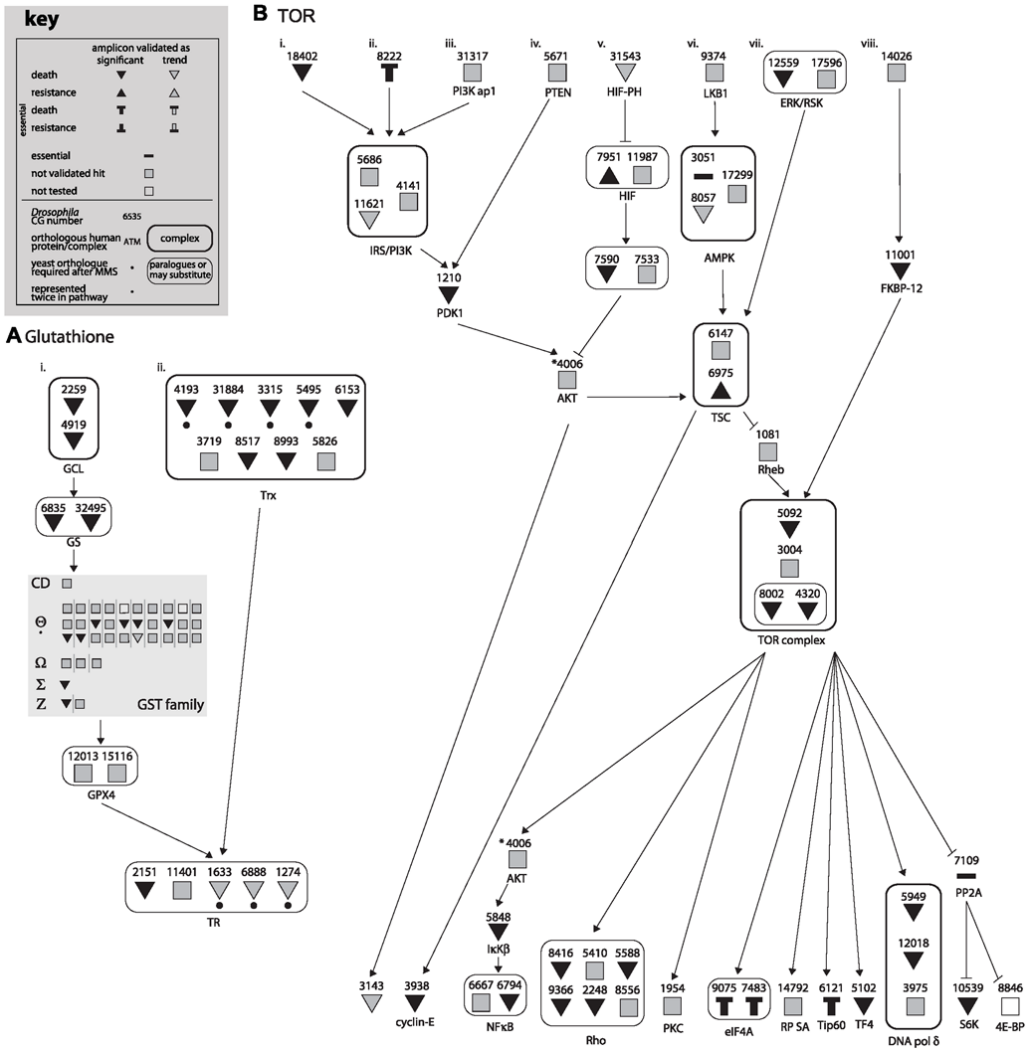
Glutathione and TOR pathways utilized for MMS survival. CG numbers are given for each *Drosophila* pathway component, as well as the protein names or complex names for their human orthologues. Pathway entry points are noted with Roman numerals at the top, and end points are at the bottom. A key for the following symbols is provided. Symbols encircled with thick lines represent proteins that act together or in a complex, while symbols encircled with thin lines represent paralogues or proteins that may substitute for one another. Proteins found to affect MMS survival are noted as down (death) or up triangles (resistance). Statistically significant proteins are indicated with black triangles, while trend hits are indicated with grey triangles. Essential genes are noted with a thick bar and any with downwards or upwards pointing boxes were also validated as conferring death or resistance, respectively, to MMS upon knock-down. Shaded squares are proteins not found to be hits after validation, and open squares were not tested in our validation. Yeast orthologues previously found to be required for MMS survival [3] are noted with a dot under the symbol. (A) Glutathione (an expanded list of GST family members is given in Table S4) and (B) TOR.

Assuming that biological function is a more informative measure of damage response than a requirement of individual genes, we therefore endeavoured to identify those MMS survival proteins within known signaling, metabolic, and enzymatic pathways. Using both *a priori* knowledge and KegArray, we identified 13 pathways that together included 41 MMS survival proteins (Figures 1, 2, 3 and Figures S2, S3, S4, S5), among them, five DNA repair pathways (Base Excision Repair; Nucleotide Excision Repair; Mismatch Repair; Homologous Recombination Repair; and RECQ). Many of these pathways have a statistically significant number of MMS survival proteins (Table S5), and this is without accounting for pathway “branching” or “subdivision.” Considering the likely role of these pathways in MMS survival, we used an RNAi assay method, which is both more sensitive and stringent than the original screen [18], to determine whether other members of the 13 identified pathway also affected MMS survival (Table S1). By individually interrogating the 346 pathway members of the 13 pathways that were not identified in the original screen, an additional 105 MMS survival genes were discovered. This observation significantly enriched the number of MMS survival genes in each of the 13 pathways (Figures 1, 2, 3 and Figures S2, S3, S4, S5) and provides additional support to the hypothesis that each of the identified pathways are involved in MMS survival. Though this result highlights the possibility of false negative screen data, compared to our previous false negative rate of 23.6% from randomly selected data, we observe a significant enrichment for false negatives within pertinent pathways ((χ^2^ p = 3.9E-5). In total we identified 146 MMS survival genes in 13 “MMS survival pathways,” encompassing 25% to 86% of all non-essential genes within each pathway (Table S4).

Using the genes we mapped to the MMS survival pathways, we then compared pathways identified from our *Drosophila* MMS screen with the analogous screen performed in yeast [14] using genes they found to be responsive to MMS. As previously noted, we did not observe a significant overlap between the two screens when comparing MMS survival gene orthologues, however, with this pathway comparison, we mapped orthologues of yeast MMS survival genes to 10 of the 13 *Drosophila* MMS survival pathways (Figures 2 and 3; Figures S2, S3, S4, S5; Table S6). The three *Drosophila* MMS survival pathways without yeast MMS survival genes either are not conserved in yeast or have almost all of the pathway components are essential for viability in yeast, thus making them refractory to MMS viability analysis (Table S5). The gene enrichment with each pathway between species is highly supportive of a conservation of processes involved in MMS survival. A notable absentee in the *Drosophila* screen, which was observed in yeast, is 3-methyladenine-DNA-glycosylase (MAG1/AlkA), the principal protection against MMS-induced DNA damage in yeast, but no direct orthologues exist in animals. One of the two glycosylase found in *Drosophila* that act at the same step in BER, namely Thd1, was found to be required for survival after MMS treatment. Furthermore, several of the pathways observed in both species, such as proteasome [19], the TOR pathway [20], and DNA repair pathways [21], have been shown to be functionally responsive to MMS in yeast. Altogether, these results suggest a conservation of pathway function, if not individual genes, in response to MMS between species.

To demonstrate that the identified *Drosophila* pathways are functioning in the expected manner in response to MMS, five were selected for further examination – base excision repair (BER), DDR, glutathione metabolism, proteolysis by proteasomal degradation (proteasome), and the TOR pathway. Two of these pathways, BER and DDR, are expected to play a role in MMS survival [9], while the others were selected for their apparent breadth of function and their non-canonical role in damage survival. For each pathway, an appropriate functional assay was chosen, and when possible, an appropriate upstream MMS survival protein within that pathway was knocked-down (Figure S6A) to demonstrate modulation of the MMS induced response.

### Functional conservation of MMS survival pathways in *Drosophila* and mouse

**Figure 4:**
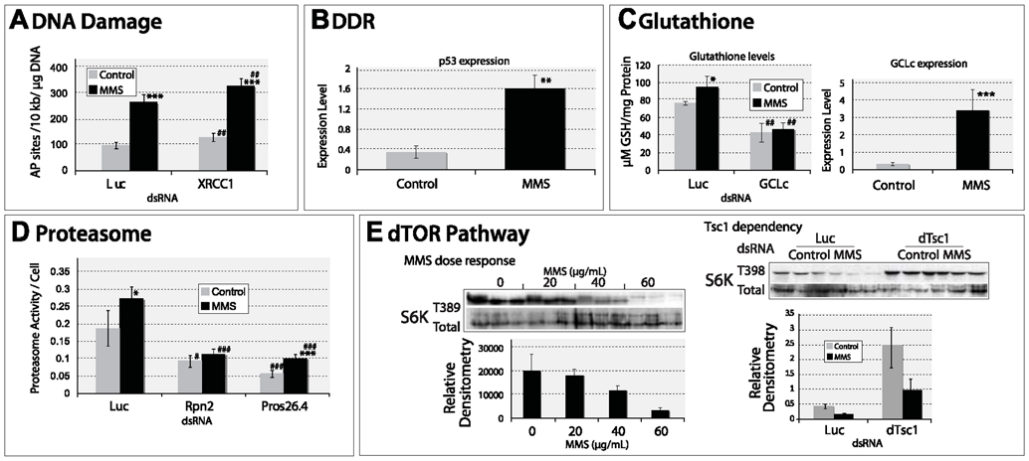
An examination of the functionality of five of pathways utilized in MMS survival by *Drosophila* cells. (A) MMS exposure results in an increase in AP sites. dsRNA knock-down of the BER component XRCC1 increases the quantity of AP sites observed following MMS exposure. (B) MMS exposure results in increased p53 expression by quantitative real-time PCR analysis; expression level is provided as fold change compared to an endogenous control (CG6905). (C) MMS results in an increased amount of total glutathione, and this increase is dependent upon the rate limiting glutathione metabolizing enzyme glutamate cysteine ligase (GCLc). The intracellular glutathione concentration is expressed as mmoles/L normalized per mg protein. This GCLc dependency correlates with the increased level of GCLc expression observed following MMS exposure. (D) Proteasome activity is increased following MMS exposure in a manner that is dependent upon proteasome components Rpn2 and Pros26.4. Proteasome activity is expressed as units of activity/cell, normalizing the activity to the number of cells using a parallel viability assessment. (E) MMS exposure results in a dose-dependent decrease in the phosphorylation of the dTor component p70S6K. This decrease is dependent upon the negative regulator of dTor, Tsc1. Statistically significant differences between unexposed and MMS exposed cells are denoted (*; **; ***; with p-values of <0.05; <0.01; and <0.001 respectively) as are differences between Luc control and each dsRNA knock-down (#; ##; ###; with p-values of <0.05; <0.01; and <0.001 respectively).

First, we tested the two expected MMS survival pathways BER and DDR. MMS-induced DNA damage results in the production of apurinic/apyrimidic (AP) sites, a DNA damage typically repaired by BER [9]. We therefore quantified the amount of AP sites per microgram of genomic DNA following MMS treatment and observed a statistically significant increase compared to control (Figure 4A; p≤7.6E-8). Knock-down of the BER component XRCC1 resulted in an increased amount of AP sites in and of itself (p≤3E-3), but following MMS treatment, the amount of AP sites in the absence of XRCC1 was increased further (Figure 4A; compared with untreated XRCC1 knock-down, p≤3.3E-8; compared to MMS treated luciferase (Luc) control, p≤3.7E-3); MMS therefore produces the expected form of DNA damage to which BER appropriately responds. p53, a central component of the DDR pathway, is regulated at expression, protein stabilization, and posttranslational modification levels. As expected, p53 gene expression increased in response to MMS exposure (Figure 4B; p≤1.6E-3).

We then examined three MMS survival pathways that are not part of the canonical DDR and DNA repair process: glutathione metabolism, proteasome, and the TOR pathway. An increased activity of the glutathione synthesis pathway following MMS exposure was demonstrated by measuring total glutathione content per milligram of protein lysate (Figure 4C; p≤3.4E-3), as well as by examining the expression of the rate-limiting enzyme for glutathione synthesis, GCLc [22] (p≤8.8E-4). Therefore, as expected, knock-down of this same protein, GCLc, significantly reduced the total amount of glutathione present compared with control (p≤3.4E-3), but its knock-down also prevented the cells from increasing the level of glutathione in response to MMS exposure (Figure 4C). Since glutathione synthesis is considered to be an oxidative stress response, we demonstrated that MMS resulted in a dose-dependent increase in the level of 8-oxoguanine, a DNA lesion normally associated with oxidative damage (Figure S7 and Text S1). Thus it appears that MMS results in not only alkylation damage but also damage from oxidative stress.

For the analysis of the proteasome degradation pathway, we measured proteasome activity and found that it increased following MMS treatment (Figure 4D; p≤4.2E-2). Protein knock-down of the proteasome components Rpn2 or Pros26.4 significantly reduced proteasome activity compared to cells without knock-down that were either unexposed (Rpn2: p≤1.7E-2; Pros26.4: p≤3.7E-3) or exposed to MMS (Rpn2: p≤1.4E-4; Pros26.4: p≤7.0E-5). Following Rpn2 knock-down, cells were unable to mount a detectable increase in proteasome activity following MMS exposure, although we were unable to demonstrate this for Pros26.4 knock-down (p≤1.7E-3). Overall, it appears that MMS exposure results in increased proteasome activity.

Finally, to investigate TOR pathway activity, S6K phosphorylation status was monitored. TOR is a kinase that phosphorylates S6K to promote growth through ribosome biogenesis and is negatively regulated by the tumor suppressor protein Tsc1 [23]. MMS exposure resulted in a dose-dependent decrease in S6K phosphorylation, suggesting an inhibition of TOR activity (Figure 4E). This MMS-induced decrease in S6K phosphorylation was dependent on Tsc1 (Figure 4E). MMS exposure therefore elicited a down-regulation of the growth promoting TOR pathway, suggesting that this pathway is also a coordinated component of DDR similar to observations in yeast [20]. This supports a previously published observation by Matsuoka *et al.* [4], who showed that some components of the mammalian TOR pathway are phosphorylated following ionizing radiation exposure. In conclusion, the results of these functional assays validate the identification of the “MMS survival pathways” by RNAi screening and that the functionality of these pathways, or lack thereof, affects MMS survival.

**Figure 5:**
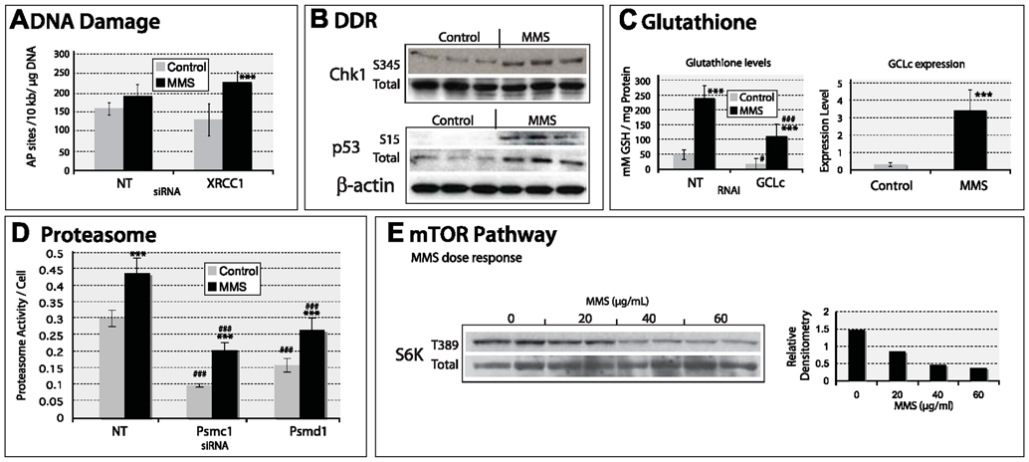
Functional conservation in mouse embryonic fibroblasts of five pathways found to be utilized for MMS survival in *Drosophila* cells. (A) MMS exposure results in an increase in AP sites, siRNA based XRCC1 knock-down results in an increased quantity of AP sites following MMS exposure. (B) MMS exposure results in phosphorylation of CHK1 and p53 and in an accumulation of total p53 levels. (C) MMS exposure results in an increased amount of total glutathione and this increase is partially dependent upon GCLc. The intracellular glutathione concentration is expressed as mmoles/L normalized per mg protein. This GCLc dependency correlates with the increased level of GCLc expression observed following MMS exposure. (D) Proteasome activity is increased following MMS exposure in a manner that is dependent upon proteasome components Pmsc1 and Psmd1. Proteasome activity is expressed as units of activity/cell, normalizing the activity to the number of cells using a parallel viability assessment. (E) MMS exposure results in a dose-dependent decrease in the phosphorylation of p70S6K. Statistically significant differences between unexposed and MMS exposed cells are denoted (*; **; ***; with p-values of <0.05; <0.01; and <0.001 respectively) as are differences between NT control and each siRNA knock-down (#; ##; ###; with p-values of <0.05; <0.01; and <0.001 respectively).

Having thus identified 13 *Drosophila* MMS survival pathways, we went on to investigate their functional conservation in mammals. Using primary mouse embryonic fibroblasts (MEFs), we examined the same five pathways that were modulated following MMS exposure in *Drosophila*. In general we observed comparable results in MEFs to *Drosophila* (compare Figures 4 and 5). MMS increased the amount of AP sites in MEFs (Figure 5A); in the absence of XRCC1, however, we observed a significant increase in AP sites following MMS exposure (Figure 5A; p≤3.7E-4). For DDR in MEFs, we assessed the phosphorylation status of Chk1 (a kinase that, once phosphorylated, phosphorylates p53) [24], total p53 protein, and the phosphorylation status of p53 (Figure 5B), not to examine p53 expression levels, but to obtain a result equivalent to that of *Drosophila* result: that DDR is activated by MMS exposure. Also, similar to the observation in *Drosophila* cells, MEFs had increased proteasome activity following MMS treatment (Figure 5D; p≤2.0E-4); knock-down of either proteasome component, PSMC1 or PSMD1 (orthologues to Rpn2 and Pros26.4, respectively), resulted in decreased proteasome activity following MMS exposure (PSMC1: p≤8.1E-5; PSMD1: p≤4.1E-4). These results are comparable with *Drosophila*: it is clear that proteasome activity is responsive to MMS in MEFs. It should also be noted that, though we were able to demonstrate the same dose-dependent decrease in S6K phosphorylation following MMS exposure in MEFs as seen in *Drosophila* cells (Figure 5E) and that knock-down of TSC1 disrupted this MMS-induced effect, the disruption was not as evident as observed with *Drosophila*, probably due to inefficient knock-down of the TSC1 protein in MEFs (data not shown). Having observed the functionality of these pathways in response to MMS and the ability of the five siRNA to modulate the MMS response of their respective pathway, we also examined the effect of each knock-down on MMS survival in MEFs. Three of the protein knock-downs, GCLC, PSMC1 and PSMD1, affected MMS survival in MEFs (data not shown). Overall, it would appear that *Drosophila* could be used to accurately predict MMS survival pathways in mouse.

### Utilization of the MMS survival pathways in response to Temozolomide

Given that MMS causes alkylation damage, it would be reasonable to suppose the identified biological pathways are generally involved in the response to other alkylating agents. Temozolomide is an alkylating agent used clinically in the treatment of cancer [12],[25]. It has already been demonstrated that BER and TOR are necessary for Temozolomide survival [12],[25]. Temozolomide causes DNA damage by increasing the number of AP sites, and similar to our MMS results, inhibition of BER increases sensitivity to temozolomide [12]. It has also been reported that rapamycin inhibition of the TOR pathway increased cellular sensitivity to temozolomide [25]. To further demonstrate the utilization of MMS survival pathways in response to temozolomide, we examined the DDR pathway, glutathione levels, and proteasome activity in MEFs and observed the same responses as observed following MMS exposure (Figure S8). Further, using HEK293 cells, we observed that both MMS and temozolomide exposure repressed Notch reporter activity (Figure S9 and Text S1). Absence of functional Notch protein has been shown to repress the activity of a downstream transcriptional activator (RBP-Jκ) [26], therefore this result demonstrates a functional Notch pathway response in alkylation damage exposure. Taken together, these data suggest a functional conservation of the MMS survival pathways responses with other similarly acting agents.

### Network integration of MMS survival pathways

**Figure 6:**
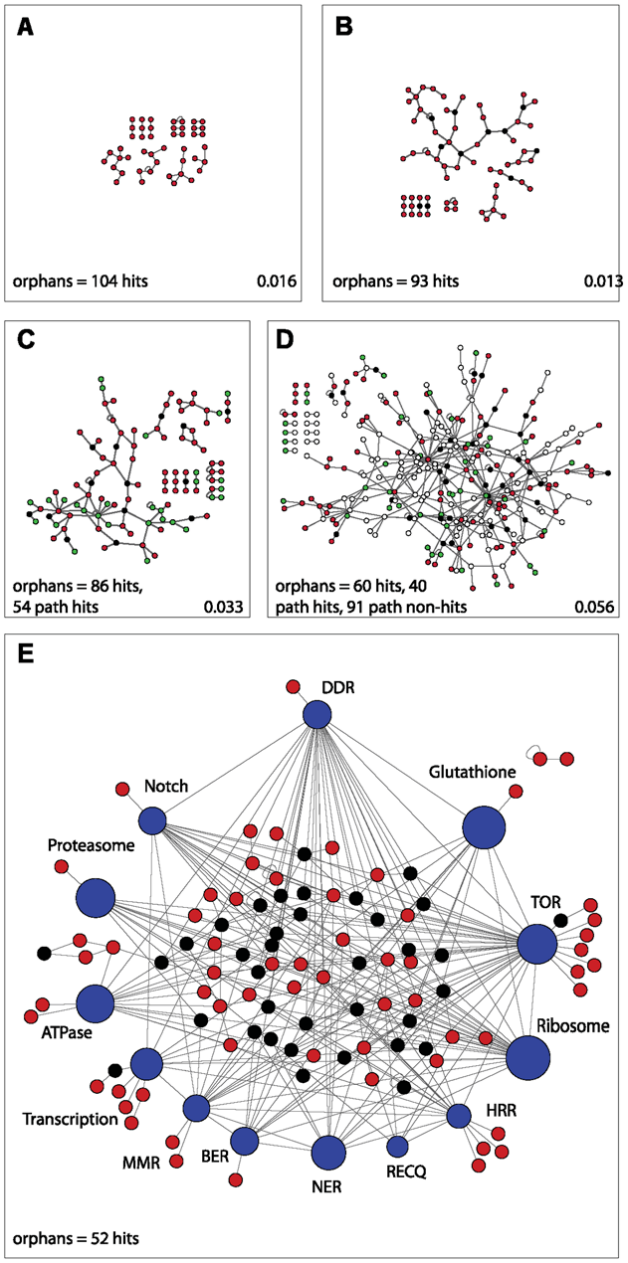
Protein network of MMS survival proteins reveals an integrated, highly connected MMS survival network. (A) 150 of the validated MMS survival proteins (red nodes) are represented in the known *Drosophila* interactome, with protein:protein interactions represented by grey edges; 104 of these validated hits are “orphans,” not connected to other proteins. (B) Nodes and orphans from (A), including 11 proteins essential for viability (black nodes) that are connected to two or more validated hits, improves the connectivity of the DNA damage survival network. In this network, only 93 hits are orphans and a larger network of 36 proteins, containing 29 validated hits, is observed. (C) Nodes and orphans from (B), including additional pathway hits not found in screen (green nodes). In this network, 86 screen hits and an additional 54 pathway hits are orphans and a larger network of 63 proteins, containing 33 validated screen hits, 20 additional pathway hits and 10 essential proteins. (D) Nodes and orphans from (C), including all non-hit members of the 13 MMS survival pathways (open nodes). In this network, 60 screen hits and an additional 40 pathway hits are orphans and a larger network of 247 proteins, containing 79 validated screen hits (26 of which are within pathways), 35 additional pathway hits, 101 non-hit pathway proteins, and 32 essential proteins. (E) Network of proteins validated to be required after MMS treatment (red nodes), proteins essential for viability (black nodes), and 13 pathways (blue pathway nodes) validated to be involved in MMS survival. All proteins in each pathway, regardless of being a hit, are represented in its metanode, with node size representing protein number. After all pathway components are used to create the network, only 54 proteins are orphan, and 96 proteins are contained within the main interconnected network. Network connectivity coefficients are given at the lower right-hand corner for (A–D).

Of the 307 identified MMS survival genes (202 validated screen hits plus 105 from pathway analyses), we were able to assign 146 to 13 pathways (Figures 1, 2, 3 and Figures S2, S3, S4, S5). Because 161 MMS survival protein remain unassigned, we re-examined the inter-relationship between the identified MMS survival proteins using the currently available *Drosophila* protein:protein interactome (PPI) map [27]–[29]. To measure the connectivity among the MMS survival genes, we took the largest connected components of the PPI network and discarded the other, smaller components. The largest connected component contained 7364 of the 7504 proteins in the original PPI network. Taking the 202 original validated MMS screen hits as an unbiased starting point, we determined the number of proteins that were directly connected to one another, compared to a random set of the same number of proteins from the PPI, and observed a significant enrichment (Figure 6A and Table S7; p≤2.1E-10). Similar results were obtained for other assessments of network connectivity (Table S7). Because the MMS screen hits had more connections on average than a set of random genes, which might have biased the above analysis, we also compared the connectivity of the MMS screen hits in the real PPI network with the connectivity of the same set of proteins in a randomly rewired PPI network. Degrees were preserved in the random rewiring process. As shown in Table S7, the MMS screen hits have statistically significantly more direct interactions than would be expected in a randomly rewired network (p = 0.01). On the other hand, the randomized network had smaller average distance and higher global efficiency than the real network, which could be attributed to the well-known property of real-world networks: they usually have slightly longer average distances (and correspondingly, lower global coefficients) than their degree-preserving randomly rewired counterparts [30].

Because we were unable to assess the effect of essential proteins (proteins whose knock-down resulted in cell death regardless of treatment) on MMS survival, we repeated the connectivity analysis while including those essential proteins that were connected to two or more MMS survival proteins, since these are the ones most likely to have a functional role in MMS survival. This increased the size and significantly improved the connectivity within the resultant network (Figure 6B and Table S7; p≤1.3E-26). After pathway analysis, we identified an additional 105 MMS pathway hits, which were validated. In order to expand the network to include the proteins in these relevant pathways, these hits were then incorporated and a larger, and equally well-connected network was observed (Figure 6C and Table S7; p≤4.6E-26). Considering the apparent importance of pathways over the individual genes, we then included all components of the 13 identified MMS survival pathways and observed that the connectivity of the resultant enlarged network was significantly improved yet again (Figure 6D and Table S7; p≤4.6E-52). Similar to the analysis of the 202 validated MMS screen hits, we also compared the connectivity of the subnetworks containing the essential genes or pathway hits with that of a randomly rewired network consisting of the same nodes. Although at a lower statistical significance, the same general trend was observed (Table S7). This more inclusive network allows a view of all interactions both within and connecting to a pathway, even if the pathway components themselves are not critical to survival after MMS. To qualitatively visualize this result, each protein known to be in a pathway was grouped together and assigned to a “pathway node” (a single node within the interactome that retains the interactions of its constitutive proteins to proteins that are external to that pathway). This resulted in a highly connected interactome, or “MMS survival network” (Figure 6E), that now encompassed 179 of the 233 MMS survival proteins that are present in the PPI network (77%). Of the remaining 54 orphan proteins, 47 are only one protein removed from the MMS survival network, six are two proteins away, and only one is not connected at all. At each step in this analysis, we observed an improved network, either enlarged or better connected, not only when comparing the entire set of proteins in that network to a random set of proteins in the PPI, but also when randomizing the additional proteins added at each step (data not shown). Overall, our observations with the MMS survival network suggest that despite the general interconnectivity within protein interactomes, a pathway analysis is highly relevant because it may improve interactome connectivity and it simplifies a systems biology overview.

## Discussion

Studies performed using yeast as a model organism to predict network response to MMS reported an astounding involvement of diverse biological pathways [31]. Considering that the genes that respond to environmental stress differ between mammalian and yeast cells [17], we presumed that damage response might be different or more complex in higher eukaryotes, especially considering the presence of paralogues and thus increased genetic redundancy. We therefore performed a genome-wide, RNAi based screen with *Drosophila* cells to investigate which genes are essential for survival following damage exposure with MMS. We were able to identify and validate 307 MMS survival genes, the majority of which had not previously been associated with alkylation damage survival. Of these genes, 146 were components of 13 different MMS survival pathways. With the five pathways examined in detail, we observed that four were functionally conserved in yeast and all five conserved in mouse with regard to their utilization following MMS treatment (Figure 5). In yeast, experimental validation of response to MMS by proteasome [19], the TOR pathway [20], and DNA repair pathways [21] was previously reported. Similarly, glutathione response to MMS was observed in mammalian cells [32], and our observation of an increase in GCLc expression provides an underlying mechanism for this phenomenon. Our demonstration of a dose-dependent increase in 8-oxoguanine after MMS exposure indicates that MMS also results in oxidative stress damage, as previous studies suggested [33]. Additionally, several recent studies have demonstrated a role for the proteasome in regulating several DNA repair pathways (reviewed in [34]), supporting our observation of increased proteasome activity in response to MMS. Thus, our screen and pathway identification have revealed a conserved set of MMS survival pathways.

Our *Drosophila* based study has provided novel insights to the global cellular response to alkylation damage by identifying biological pathways whose functions are required for survival after this damage. The only other analogous genome-wide, loss-of-function screen for MMS survival genes was performed in yeast [14]. That study highlighted the general biological processes required for MMS survival based on gene ontology and integrated the identified proteins into a disorganized network [14]. Considering that pathways, whether signaling, metabolic, or enzymatic, have long been identifiable entities, it is logical to consider them as units within a network. Thus our experiments focused on identifying pathways required upon exposure to damage and validating the biological responsiveness of pathways following this damage exposure. Our results confirmed that these biological pathways are indeed functional in yeast, *Drosophila*, and mouse cells and therefore functional contribution of these biological pathways are pertinent to damage response in a network representation.

In addition to the functional conservation of the survival pathway in response to alkylation damage, these same biological pathways appear to have roles in response to other types of damages. It is interesting to compare our results with an elegant study by Matsuoka *et al.* [4], which identified proteins that are phosphorylated following ionizing radiation in human cells. Their study identified proteins that are components of nine of our 13 MMS survival pathways, including four of the DNA repair pathways, DDR, mTOR, proteasome, basal transcription, and ribosome. These results suggest that different types of damage, not just alkylation damage, may utilize different components of a DNA damage survival network in a functionally conserved manner and reemphasize the functional conservation of pathways, if not the individual genes, between species.

Our emphasis to reorganize the MMS survival network based on pathways is to facilitate the observation of biologically relevant interactions. Often protein:protein interaction networks may appear chaotic, but may be interrogated for simple sub-networks associated with protein(s) and pathways of interest [21]. However, when working with pleiotropic responses that encompass so many different biological processes, such as DDR, a chaotic network representation appears non-intuitive (Figure 6D). Thus, the integration of pathways within the conceptual framework of systems biology networking is logical. An additional advantage of including pathways is highlighted by our demonstration of MMS survival protein enrichment by detailed examination of pathways. Even with this detailed analysis and convincing evidence that the pathways were indeed functioning as expected, we were not able to assign every protein within each pathway as an “MMS survival protein.” There are many possible explanations for this, but nonetheless, considering the pathways as a whole provide a framework within the network that highlights novel interactions, cross-talk, and identified proteins not mapped to a canonical pathway, but present within the network, would unlikely be observed in a “chaotic network view” and encourages their investigation. This approach is similar to computational clustering of networks based on signaling pathways using interactome datasets [35], but our approach includes both identified proteins and pathway components. Our representation is simplified, using a single node to represent the entire pathway rather than a complex display of interactions for every component in a pathway [36]. This simplified pathway inclusive representation reveals a highly organized network, consistent with the requirement of each pathway for cellular survival (Figure 6E), and provides an effective strategy to integrate modular components into the network [37] and thereby inferring biological properties [38].

The interconnectivity between the survival pathways (Figure 5E) suggests potential pathway cross-talk. If such cross-talk exists, it would be highly pertinent to cancer therapy. Recent studies have demonstrated the utility of a global level analysis, allowing identification of altered pathway function in complex diseases such as the Notch pathway in pancreatic cancer [39] and similarly the DDR pathway in breast and colorectal cancers [40]. Considering our identification of Notch, TOR, DDR, and the proteasome as “survival pathways,” all of which are currently being explored as targets for cancer therapy [41],[42], our identified survival network would suggest the possibility of combining pathway-specific pharmacological agents in cancer therapy. Some of the pathway connections and potential cross-talk represented in our survival network (Figure 6E) have already been observed. For example, protein phosphatase, PP2A, a downstream component of the TOR pathway [23], interacts with the DDR component to regulate phosphorylation of ATM and ATR [43] and vice versa [4]; DDR interacts with BER via CHK2 and XRCC1 [44]; Notch interacts with DDR via Mastermind and p53 [45]; the glutathione pathway interacts with the nucleotide excision repair pathway (NER) [46]; and the proteasome interacts with various DNA repair components [34],[47]. Together, it would appear that our model of an integrated network of conserved damage survival pathways is both valid and biologically relevant.

In conclusion, we have identified a network of pathways that have a functional role in damage response by affecting viability; we also demonstrated the functional conservation between species of the MMS survival pathways. By considering the protein interactions between the MMS survival proteins and by incorporating the MMS survival pathways, a highly interconnected damage survival network is observed that encompasses at least 58% of the identified MMS survival proteins directly. This interconnectivity suggests a strong functional interrelationship between constitutive components of the survival network and the possibility of pathway cross-talk and coordination at a level greater than just their instigation by the DDR pathway. Although these MMS survival pathways have already been implicated in MMS damage response, we have identified these seemingly disparate pathways in a single screen, and with the network analysis, this lends to direct connection of these pathways in response to damage. The pleiotropic effects of alkylation damage therefore require a wide-variety of functioning pathways in order for the cell to survive.

## Methods

### Tissue culture

*Kc_167_* cells were grown in Schneider medium (Invitrogen, Carlsbad, CA) supplemented with 10% heat inactivated fetal bovine serum (65°C, 10 minutes), penicillin and streptomycin at 22°C in a humidified chamber.

Primary MEFs were obtained by harvesting 14.5 day C57BL/6 embryos as previously described [48]. Briefly, fetal liver and head were removed and the remainder of the embryo mechanically disaggregated in plating medium. A suspension of single-cells was plated out in Dulbecco's Modified Eagle's Medium (DMEM) supplemented with 10% fetal calf serum, 2 mM glutamine, 100 U/mL penicillin, and 100 µg/mL streptomycin. MEFs were grown for two passages before freezing aliquots. Aliquots were taken and expanded as needed for each experiment.

### Genome-wide viability screen following MMS exposure

Methods were adapted from [49], with 1.2×10^4^ *Kc_167_* cells suspended in 10 µL of serum-free Schneider medium (Invitrogen) added to each well of sixty-three 384-well plates. Each well contains about 0.25 µg of a double-stranded (ds) RNA, with 22,915 individual dsRNA represented in the whole library (Version 1 dsRNA). Cells were incubated for 1 h at 22°C, allowed phagocytic uptake of the dsRNA, then 20 µL of serum (15% heat-inactivated fetal bovine serum) containing medium were added and the plates incubated for a further 72 h. Medium was exchanged for fresh serum containing medium, with or without 0.004% (w/v) of MMS (Sigma-Aldrich, St. Louis, MO). Following 72 h additional incubation, medium was removed and the number of viable cells assessed using pre-diluted Celltiter-Glo per manufacturers instructions (Promega, Madison, WI). Screens were performed in duplicate, with the replicate screen initiated on a different day.

### Production of dsRNA

dsRNA were produced similarly to previously described [8],[18]. Briefly, dsRNA was synthesized using cDNA prepared by gene specific amplification of reverse transcribed cDNA. For cDNA preparation, RNA was harvested from adult *D. melanogaster* using Trizol reagent. For gene specific amplification, primers designed by the DRSC to target genes of interest were custom synthesized with overhanging T7 promoter sequences (Invitrogen). DRSC validation primer sequences were obtained from the DRSC for targeting genes of interest, and oligonucleotides were purchased (Invitrogen). First round PCR amplifications were performed using gene specific primers, and the amplified products were gel purified, using an agarose gel purification kit (Qiagen, Valencia, CA) or a 96-well gel isolation kit (Invitrogen). The purified PCR products were then used for second round amplification also using the gene specific primers; these amplified products were purified using Millipore PCR purification 96-well plate system (Millipore, Billercia, MA). The purified products were then used for dsRNA synthesis using T7 Ribomax express system (Promega) and purified using the PCR purification plate system (Millipore). The quality of purified dsRNA was verified by agarose gel electrophoresis and, following spectrophotometric quantification, stored at −20°C.

### Validation analysis

Validation of MMS survival genes was performed as previously described [18], with the exception that DRSC “validation” dsRNA (targeting a gene mRNA transcript at a different location to the library amplicon), designed by the DRSC but produced in-house, were preferentially used in this study. Briefly, dsRNA were validated in quadruplicate, with one set of plates treated with MMS and one set used as an untreated control. Plates with or without MMS with the same dsRNA were then compared.

Genes required after MMS treatment and genes whose knock-down conferred resistance to MMS were validated by validation amplicons, if available. When validation amplicons were not available, the amplicon used in the original library was used for validation. In some instances, both validation and library amplicons were used in validation experiments. In these cases, the knock-down result from the validation amplicon was preferred over that of the library amplicon, if a difference was evident. If the gene of interest was annotated to a pathway that had already been determined to be involved in MMS response, the gene was considered as a hit regardless of which version amplicon validated.

### Gene ontology

Gene ontology was retrieved from FlyBase (http://flybase.org/). Enrichment analysis was conducted using FuncAssociate (http://llama.med.harvard.edu/cgi/func/funcassociate/) for those ontologies over-represented with a p-value of less than 0.05. Those ontology categories that overlapped were consolidated.

### Westerns

Protein lysates were prepared using radioimmunoprecipitation assay (RIPA) buffer containing 5% sodium deoxycholate, 0.1% SDS, 0.1% Igepal in PBS with a cocktail of protease inhibitors (1 mM PMSF, 1 mM Sodium Orthovandate and 30 uL/mL Aprotinin (Sigma)). Protein concentration was determined using Bradford Protein Assay Reagent (Biorad, Hercules, CA). Equal amounts of protein were resolved using 10% SDS polyacrylamide gel and transferred onto a nitrocellulose membrane (Hybond-ECL, GE Healthcare Lifesciences, Piscataway, NJ). Using appropriate dilutions of primary and secondary antibodies, immunodetection of the protein was performed using the ECL plus system (GE Healthcare Lifesciences). Primary antibodies include P-Chk1 (T68) (Abcam) and total CHK1 (Abcam), P-p70S6K (T389) (Cell Signaling Technology) and total p70S6K (Cell Signaling Technology), and P-p53 (S15) (Cell Signaling Technology) and total p53 (Cell Signaling Technology).

### Apurinic sites measurement

Apurinic or apyrmidinic sites (AP) that were generated following DNA damage were measured using a colorimetric assay kit for DNA damage quantification (Oxford Biomedical Science, Oxford, MI), following manufacturer's protocol. Briefly, DNA was isolated from the experimental cells using a Genomic DNA isolation kit (Oxford Biomedical Science). Equal amounts of DNA were then labelled using a biotinylated aldehyde reactive probe. Labelling was followed by purification and colorimetric quantification using streptavidin-horse radish peroxidase (HRP) conjugate and HRP-dependent substrate supplied with the kit. The aldehyde reactive probe labelled DNA standard, supplied by the kit, was used to determine the number of AP sites per 100 kilobase pairs of DNA in the experimental samples.

### Glutathione measurement

Intracellular glutathione levels were measured using a colorimetric assay kit for glutathione (Oxford Biomedical Science), following manufacturer's protocol. Briefly, cells from RNAi experiments were resuspended in ice-cold PBS and homogenized using a sonicator; the lysate was cleared by centrifugation and the protein concentration of the supernatant was determined using Bradford's reagent (Biorad). For estimation of glutathione, equal amounts of protein lysate were de-proteinized using metaphosphoric acid (MPA) with the final concentration of MPA adjusted to 5%. The precipitated proteins were cleared by centrifugation at 4000 g for 10 min at 4°C, and the supernatant was used for the assay. Reduced glutathione (Sigma) was used as a standard, and samples were arrayed in a 96-well plate. A two-step reaction was conducted, thioesterification of intracellular thiols using 4-choloro-1-methyl-7-trifluoromethyl-quinolinium methylsulfate followed by alkaline conversion of glutathione-thioester to chromophoric thione, followed by detection of total glutathione by absorbtion at 400 nm.

### Quantitative Reverse-Transcriptase PCR

dsRNA was used to target a gene of interest; this dsRNA was the same used to validate the genes of interest by knock-down. The level of RNAi mediated silencing of gene expression was monitored by quantitative real time RT–PCR using QuantiTect SYBR Green RT-PCR kit (Qiagen GmbH) and an ABI 7500 Real Time PCR System (Applied Biosystems, Foster City, CA). For these experiments, 18.61×10^4^ *Kc_167_* cells were dispensed into a 24-well tissue culture plate containing 6.2 µg of dsRNA per well. Following 1 h incubation in a serum-free condition to allow the uptake of dsRNA, serum was replenished to a final concentration of 10%. On Day 3, RNA was isolated using RNeasy Mini kit (Qiagen) and quantified using an ND-1000 Spectrophotometer (Nanodrop, Wilmington, DE). For the PCR amplifications, distinct primers that were not encompassed within the dsRNA used to target the gene were used. For gene expression analysis following MMS exposure, RNA was isolated on Day 4. For experiments with *Kc_167_* cells, CG6905, the expression of which remained unaltered following MMS exposure, was used as an endogenous control. For experiments with MEFs, the isolated RNA was reverse transcribed using an ImProm-II Reverse Transcription System (Promega) and the cDNA was used with TaqMan Gene Expression Assay kit for GCLc, purchased from (Applied Biosystems, CA) and TaqMan Universal Master Mix (Applied Biosystems). Mouse β-Actin TaqMan Gene Expression Assay kit was used as an endogenous control. The level of gene expression was determined using ▵▵C_t_ method [50].

### Proteasome measurement

Experiments were performed in 384-well plates using the same layout and timing as described for the RNAi validation experiments, [18], except the proteasome activity was measured on Day 4 of the experiment, 24 h after MMS exposure. For proteasome measurement, a Proteasome-Glo assay kit was used (Promega), as per instructions. To normalize the proteasome activity with cell density, a parallel experiment was performed in a separate 384-well plate, and the cell density was estimated using CellTiter-Glo (Promega) as described above. Proteasome activity was then normalized to the relative number of cells present.

### Orthologue identification

Orthologues of *Drosophila* genes of interest in human, mouse, and yeast, were obtained from Ensembl49 (http://ensembl.org/).

### siRNA targeting of MEFs

SMARTPool siRNAs were purchased from Dharmacon RNA technologies (Lafayette, CO). For siRNA transfection into MEFs, cells were harvested by trypsinization, washed in a serum free medium, and cell density was adjusted to 1×10^6^ cells in 100 µL of MEF-2 Nucleofector Solution (Amaxa, Gaithersburg, MD), containing 0.5 µg of siRNA, followed by transfection by electroporation using Nucleofector II Device (Amaxa). The transfected cells were seeded in either a 96-well or a 384-well tissue culture plate, as required by the experiment. For non-specific control, scrambled, non-targeting siRNA was used. The fluorescent probe siGLO red (Dharmacon) was used to monitor the efficiency of siRNA uptake, and the efficiency of protein knock-down was determined by western blot analysis (Figure S3B). Additional validation for SMARTPool siRNA experiments were performed using a minimum of four independent duplex siRNA for each gene (Figure S10).

### Pathway identification

Pathway analysis was conducted first by *a priori* identification of protein/pathway relationships and KegArray, yielding from which we included DNA damage response, glutathione metabolism, the TOR pathway, proteasome, and DNA repair pathways. For a systematic analysis on the MMS hits, KegArray (with default settings) was used (http://genome.jp/download/), yielding Notch signaling, ATPase, basal transcription, ribosome, proteasome and glutathione metabolism. These pathways were examined for the number of hits identified in the pathway versus the number of total pathway components. Pathways that we included from prior knowledge only were not top pathways retrieved by KegArray due to their relatively small size or completeness in the KEGG database in fly.

### Interactome analysis

Protein interactome data were obtained from IntAct [27] (http://www.ebi.ac.uk/intact/), the Database of Interacting Proteins [28] (DIP, http://dip.doe-mbi.ucla.edu/) and the Biomolecular Interaction Network Database [29] (BIND, http://bond.unleashedinformatics.com/). Data from the three databases were combined into a single interactome, using the CG number of each gene as the identifier of the protein. Interactomes were visualized using Cytoscape 2.6 (http://www.cytoscape.org/). Pathway nodes were created external to Cytoscape by renaming all nodes representing protein in the pathways with the name of the pathway. If a protein was found in multiple pathways, it is represented in all relevant pathway nodes. Interactions between pathways were trimmed if the only interaction between them also existed within both pathways. For instance, if a protein:protein interaction occurs between two members of the NER pathway, and these two proteins also exist in the BER pathway, then the interaction is more likely to be pathway specific and not cross-talk between pathways and was therefore removed.

### Connectivity analyses

Four measurements of connectivity were made: (1) Counting the number of pairs of MMS survival proteins that directly interact in the interaction network, (2) computing the average geodesic distance (i.e., the number of edges in a shortest path) between each pair of MMS survival proteins in the interaction network [51], (3) the global efficiency of the network [51], and (4) the clustering coefficient [51]. The number of direct interactions provides an intuitive measure of the connectivity of a subnetwork, while the average distance measures the global connectivity of the sub-network. Global efficiency provides a similar measure to average distance, but allows for disconnected components. The global efficiency was measured by 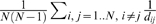, where *n* is the number of vertices in the network and *d_ij_* is the geodesic distance between vertices v_i_ and v_j_. The clustering coefficient is a measure of local connectivity of the network. For each vertex v_i_, let g_i_ be the subnetwork that consists of direct neighbours of v_i_ (excluding v_i_ itself) and the edges between them. The clustering coefficient of v_i_ was measured by the total number of edges in g_i_ divided by the maximum number of edges that could possibly exist in g_i_. The clustering coefficient of a network is given by the average of the clustering coefficient of each vertex, with a high clustering coefficient indicating a distinction between a real network from a random one. To assess the statistical significance of each measurement, the same number of proteins or proteins pairs, as appropriate, were randomly sampled from the PPI or PPI subnetwork. This sampling was repeated 1,000 times to estimate a p-value that the difference could be expected by chance.

### Statistical analyses

To determine if knock-down of genes resulted in increased sensitivity to MMS, raw data obtained from the viability assays was normalized and statistically analyzed as described previously [18]. A T-test was performed between normalized quadruplicates to determine the significant difference between treated and untreated wells. Percent control survival with MMS treatment was then estimated for each experimental gene knock-down as described previously [18], and a second T-test was performed on the percent of viability as compared with luciferase control within each plate as described before [18]. From these analyses, significant hits were selected as death hits if there was no greater than 55% viability in treated wells as compared to untreated if a p-value of less than 0.05 resulted from at least one of the T-tests. For those genes with an essential phenotype of less than 40% viability of non-targeting dsRNA, more stringent requirements were made on the viability effect after MMS treatment, such that 30–40% viability when untreated needed 15% viability after MMS treatment, 20–30% needed 10% after treatment, 10–20% needed 5% after treatment, and 0–10% was too dead to determine if MMS had an effect. Genes were considered “trend death hits” if they exhibited less than 55% viability after treatment but did not have a p-value with significance or if they exhibited 55–65% viability after MMS treatment with a significant p-value. Knock-down of genes resulting in resistance to MMS were confirmed as those with greater than 85% viability after treatment as compared to untreated wells, with one of the two p-values of less than 0. 0001 and viability after no treatment to be at least 60% that of non-targeting knock-down.

To determine if a gene was essential for viability, a T-test was performed on untreated wells comparing values for non-targeting dsRNA against luciferase and dsRNA targeting the gene. If the targeting dsRNA resulted in less than 70% viability of luciferase with a p-value of less than 0.00001, the gene was deemed essential.

Comparisons between predicted and observed numbers of MMS survival genes between yeast and *Drosophila* studies were done by a standard G-test [52]. The G-test is equivalent to a contingency Chi-square test but allows for classes with zero events.

To determine protein enrichment within a pathway where there is an unbalanced distribution of data between the numbers of proteins within a pathway compared to the total number of genes within a genome, a Fisher's Exact test was employed [53].

For connectivity measurements, except in the case of clustering coefficient, p-values were estimated using a Z-test, given that the connectivity measurement for the random subnetworks approximately follows a normal distribution (Table S6). The p-value for the clustering coefficient measurement was estimated by simply counting the frequency that the clustering coefficient of a randomly sampled sub-network was at least as high as that of the real sub-network.

## Supporting Information

Figure S1 The MMS dose response as measured by cell survival. Viability of *Drosophila Kc_167_* cells following exposure to increasing dose of MMS determined using CellTiter-Glo (relative light units (ordinate) for increasing dose of MMS (abscissa)).
(0.54 MB EPS)

Figure S2 Four of the eight additional pathways utilized for MMS survival. CG numbers are given for each *Drosophila* pathway component, as well as the protein names or complex names for their human orthologues. Pathway entry points are noted with Roman numerals at the top, and end points are at the bottom. A key for the following symbols is provided. Symbols encircled with thick lines represent proteins that act together or in a complex, while symbols encircled with thin lines represent paralogues or proteins that may substitute for one another. Proteins found to affect MMS survival are noted as down (death) or up triangles (resistance). Statistically significant proteins are indicated with black triangles, while trend hits are indicated with grey triangles. Essential genes are noted with a thick bar and any with downwards or upwards pointing boxes were also validated as conferring death or resistance, respectively, to MMS upon knock-down. Shaded squares are proteins not found to be hits after validation, and open squares were not tested in our validation. Yeast orthologues previously found to be required for MMS survival [3] are noted with a dot under the symbol. An example of average percent of untreated control survival of validated hits is shown next to each pathway, though this may not represent the actual control for each data point within the graph. Error bars are the standard deviation of quadruplicates. Survival of control cells with dsRNA targeting luciferase is shown in an open bar, protein knock-down that resulted in a significant difference in MMS survival from this control are shown in black (death) or stripped (resistance), and those with a trend effect are shown in grey. A complete list of these proteins and their human and yeast orthologues is given in Table S6. (A) Nucleotide Excision Repair. (B) Mismatch Repair. (C) Homologous Recombination Repair. (D) RecQ Helicases.
(1.40 MB EPS)

Figure S3 Two of the eight additional pathways utilized for MMS survival. CG numbers are given for each *Drosophila* pathway component, as well as the protein names or complex names for their human orthologues. Pathway entry points are noted with Roman numerals at the top, and end points are at the bottom. A key for the following symbols is provided. Symbols encircled with thick lines represent proteins that act together or in a complex, while symbols encircled with thin lines represent paralogues or proteins that may substitute for one another. Proteins found to affect MMS survival are noted as down (death) or up triangles (resistance). Statistically significant proteins are indicated with black triangles, while trend hits are indicated with grey triangles. Essential genes are noted with a thick bar and any with downwards or upwards pointing boxes were also validated as conferring death or resistance, respectively, to MMS upon knock-down. Shaded squares are proteins not found to be hits after validation, and open squares were not tested in our validation. Yeast orthologues previously found to be required for MMS survival [3] are noted with a dot under the symbol. An example of average percent of untreated control survival of validated hits is shown next to each pathway, though this may not represent the actual control for each data point within the graph. Error bars are the standard deviation of quadruplicates. Survival of control cells with dsRNA targeting luciferase is shown in an open bar, protein knock-down that resulted in a significant difference in MMS survival from this control are shown in black (death) or stripped (resistance), and those with a trend effect are shown in grey. A complete list of these proteins and their human and yeast orthologues is given in Table S6. (A) Basal Transcription. (B) Ribosome.
(3.26 MB EPS)

Figure S4 Two of the eight additional pathways utilized for MMS survival. CG numbers are given for each *Drosophila* pathway component, as well as the protein names or complex names for their human orthologues. Pathway entry points are noted with Roman numerals at the top, and end points are at the bottom. A key for the following symbols is provided. Symbols encircled with thick lines represent proteins that act together or in a complex, while symbols encircled with thin lines represent paralogues or proteins that may substitute for one another. Proteins found to affect MMS survival are noted as down (death) or up triangles (resistance). Statistically significant proteins are indicated with black triangles, while trend hits are indicated with grey triangles. Essential genes are noted with a thick bar and any with downwards or upwards pointing boxes were also validated as conferring death or resistance, respectively, to MMS upon knock-down. Shaded squares are proteins not found to be hits after validation, and open squares were not tested in our validation. Yeast orthologues previously found to be required for MMS survival [3] are noted with a dot under the symbol. An example of average percent of untreated control survival of validated hits is shown next to each pathway, though this may not represent the actual control for each data point within the graph. Error bars are the standard deviation of quadruplicates. Survival of control cells with dsRNA targeting luciferase is shown in an open bar, protein knock-down that resulted in a significant difference in MMS survival from this control are shown in black (death) or stripped (resistance), and those with a trend effect are shown in grey. A complete list of these proteins and their human and yeast orthologues is given in Table S6. (A) ATPases. (B) Notch.
(2.47 MB EPS)

Figure S5 Average percent of untreated control survival of validated hits in each pathway represented in Figures 2 and 3. Error bars are the standard deviation of quadruplicates. Survival of control cells with dsRNA targeting luciferase is shown in an open bar as an average across all plates as a general reference. It should be noted that each plate had its own luciferase controls against which all plate values were compared. Protein knock-down that resulted in a significant difference in MMS survival from their internal plate control are shown in black (death) or dashed (resistance), and those with a trend effect are shown in grey. A complete list of these proteins and their human and yeast orthologues is given in Table S6.
(4.48 MB EPS)

Figure S6 Efficiency of RNAi mediated silencing of gene expression. (A) RNAi transfection resulted in decreased expression of target mRNA in *Drosophila Kc_167_* cells, measured by quantitative real time PCR as indicated percent control gene expression (ordinate) for the genes (abscissa) tested, with expression normalized to endogenous control (CG6905). (B) Western blot analysis for efficiency of RNAi in siRNA transfected primary mouse embryonic fibroblast cells.
(0.74 MB EPS)

Figure S7 MMS exposure results in a dose-dependent increase in 8-oxoguanine DNA modifications. The MMS-dependent increase in 8-oxoguanine is (A) observed qualitatively by microscopic examination with 8-oxoguanine containing cells observed by fluorescence in the FITC channel and this (B) can be quantified as a percentage of fluorescent within a field (ordinate) for increasing dose of MMS (abscissa).
(7.71 MB EPS)

Figure S8 Temozolomide exposure results in functional response by MMS survival pathways in mouse embryonic fibroblasts. (A) Temozolomide exposure results in phosphorylation of p53 and in an accumulation of total p53 levels. (B) Temozolomide exposure results in an increased amount of total glutathione. The intracellular glutathione concentration is expressed as units of activity/cell. (C) Proteasome activity is increased following temozolomide exposure, normalizing the activity to the number of cells using a parallel viability assessment. Proteasome activity is expressed as units of activity/cell, normalizing the activity to the number of cells using a parallel viability assessment.
(0.71 MB EPS)

Figure S9 MMS and Temozolomide exposure results in functional response by notch signalling pathway in HEK 293 cells. MMS and temozolomide exposure results in decreased luciferase activity of the notch reporter RBP-Jk, normalized for transfection efficiency in HEK 293 cells using renilla luciferase.
(0.41 MB EPS)

Figure S10 Validation of pathway functions with additional siRNA knock down in mouse embryonic fibroblasts. (A) Knock-down of GCLc with four different siRNA results in decreased GCLc expression in mouse cells, by quantitative real-time PCR analysis; expression level is provided as fold-change compared to an endogenous control (mouse β-actin). (B) MMS results in an increased amount of total glutathione, and this increase is dependent upon the rate limiting glutathione metabolizing enzyme glutamate-cysteine ligase (GCLc). (C) Knock-down of proteasome components Psmc1 or Psmd1 with four different siRNA results in decreased gene expression in mouse cells, by quantitative real-time PCR analysis; expression level is provided as fold-change compared to an endogenous control (mouse β-actin). (D) Proteasome activity is increased following MMS exposure in a manner that is dependent upon proteasome components Pmsc1 and Psmd1.
(0.81 MB EPS)

Table S1 Summary of MMS screen hits and pathway genes selected for validation. The 534 *Drosophila* MMS screen hits selected for validation analysis and 298 pathway genes tested for pathway analysis, provided with FlyBase gene number, corresponding CG number, and the dsRNA used for validation, noted by DRSC identification number, either library dsRNA or validation dsRNA. For those genes that had no validation amplicon designed or those whose library amplicon had no potential off-target effects at 19 nt, data from [18] was used (asterisks). For each dsRNA, significant death, trend death or significant resistance to MMS treatment is noted (MMS survival).
(0.94 MB XLS)

Table S2 Raw, normalized, and survival data for validation experiment. For data normalization, raw data of untreated control and MMS experiments were normalized using luciferase (Luc) and high MMS controls and statistical significance was determined as described [18].
(0.23 MB XLS)

Table S3 List of yeast MMS hits and their fly orthologues. Yeast MMS hits as determined by Begley et al. [3], and their fly orthologues that were neither a hit in our MMS screen nor in a pathway identified from the screen. Also given is whether each fly gene was essential, a resistance hit, a death hit, or a death tread after validation with an independent dsRNA.
(0.04 MB XLS)

Table S4 *Drosophila* GST family members and their involvement in MMS survival. The five GST families and their component members. Provided is the gene name, corresponding CG number, whether they validated as a significant MMS survival gene (death), a trend (death trend) or not involved in MMS survival (no). For each validation observed to have an effect, the type of amplicon, library or validation is given. It is also noted for any validated MMS survival gene whether the effect was also observed in the screen. If validation was not performed this is also noted (not tested).
(0.02 MB XLS)

Table S5 Summary of the number of MMS survival genes identified per MMS survival pathway (BER: base excision repair; DDR: DNA damage response; Proteasome; GSH: glutathione synthesis; TOR: TOR pathway; NER: nucleotide excision repair; MMR: mismatch repair; HRR: homologous recombination repair; RECQ: RecQ helicases; Transcription: basal transcription; Ribosome; ATPase; Notch: Notch signaling pathway), and the percentage non-essential genes of each pathway that this represents. Protein enrichment within each pathway compared to the number of genes that validated (202 of 13826 prior to pathway analysis and 307 of 13826 including pathway analysis) is determined using a Fisher's Exact Test (NS: Not Significant; NA: Not Applicable).
(0.51 MB PDF)

Table S6 The orthologous relationship between the MMS survival genes identified in the *Drosophila* and yeast MMS survival screens. Provided are the 13 MMS survival pathway, the gene names of each *Drosophila* component and their corresponding CG number. For each of these *Drosophila* genes, the yeast ORF is provided for any identifiable yeast orthologue and whether it was observed as being involved in MMS survival (hit) in *S. cerevisiae* by Begley et al. [3], if it was not identified (not hit) or if it is essential (essential). Where orthologues between the species are known and taking into account the yeast essential genes that could not be assayed for their effect on MMS survival, there is a clear enrichment in MMS survival genes within the majority of pathways examined between the two species. A complete list of these proteins and their human orthologues are also given.
(0.11 MB XLS)

Table S7 Connectivity analysis of MMS survival genes identified by the RNAi genomic screen and following validation analyses. Provided are the number of proteins identified, the number of those proteins that are within the protein:protein interactome (PPI), the number of direct connections between these proteins, the average distance between every possible pair of proteins within the network compared to the expected values, the global efficiency and the clustering coefficient. For each measurement the expected number of direct interactions is derived from the same number of proteins randomly selected 1000 times from the PPI. Analyses are provided for the proteins identified by the MMS screen prior to validation (Screen hits), those screen hits that actually validated (Validated hits), the validated hits as well as essential proteins that are connected to two or more hits (Validated+essential connectors), the total number of validated MMS survival proteins identified by both screen and pathway validation (Validated+pathway hits), the Validated and pathway hits and the essential proteins that are connected to two or more hits (Validated+pathway hits+essential connectors), the total number of validated MMS survival proteins and all other components of the 13 MMS survival pathways (Validated+all in pathways), and finally the total number of validated MMS survival proteins, all other components of the 13 MMS survival pathways and the essential proteins that are connected to two or more hits or pathway proteins (Validated+all in pathways+essential connectors). Analyses were also provided to compare connectivity of real and randomly rewired PPI.
(0.19 MB DOC)

Text S1 Methods for 8-oxo-guanine assay and notch reporter analysis.
(0.03 MB DOC)

## References

[1] Altieri F, Grillo C, Maceroni M, Chichiarelli S (2008). DNA damage and repair: from molecular mechanisms to health implications.. Antioxid Redox Signal.

[2] Shimada M, Nakanishi M (2006). DNA damage checkpoints and cancer.. J Mol Histol.

[3] Begley TJ, Rosenbach AS, Ideker T, Samson LD (2004). Hot spots for modulating toxicity identified by genomic phenotyping and localization mapping.. Mol Cell.

[4] Matsuoka S, Ballif BA, Smogorzewska A, McDonald ER, Hurov KE (2007). ATM and ATR substrate analysis reveals extensive protein networks responsive to DNA damage.. Science.

[5] Workman CT, Mak HC, McCuine S, Tagne JB, Agarwal M (2006). A systems approach to mapping DNA damage response pathways.. Science.

[6] Kelley R, Ideker T (2005). Systematic interpretation of genetic interactions using protein networks.. Nat Biotechnol.

[7] Ng A, Bursteinas B, Gao Q, Mollison E, Zvelebil M (2006). Resources for integrative systems biology: from data through databases to networks and dynamic system models.. Brief Bioinform.

[8] Ramadan N, Flockhart I, Booker M, Perrimon N, Mathey-Prevot B (2007). Design and implementation of high-throughput RNAi screens in cultured Drosophila cells.. Nat Protoc.

[9] Drablos F, Feyzi E, Aas PA, Vaagbo CB, Kavli B (2004). Alkylation damage in DNA and RNA–repair mechanisms and medical significance.. DNA Repair (Amst).

[10] Rydberg B, Lindahl T (1982). Nonenzymatic methylation of DNA by the intracellular methyl group donor S-adenosyl-L-methionine is a potentially mutagenic reaction.. Embo J.

[11] Hecht SS (1999). DNA adduct formation from tobacco-specific N-nitrosamines.. Mutat Res.

[12] Liu L, Taverna P, Whitacre CM, Chatterjee S, Gerson SL (1999). Pharmacologic disruption of base excision repair sensitizes mismatch repair-deficient and -proficient colon cancer cells to methylating agents.. Clin Cancer Res.

[13] Malet-Martino M, Gilard V, Martino R (1999). The analysis of cyclophosphamide and its metabolites.. Curr Pharm Des.

[14] Begley TJ, Rosenbach AS, Ideker T, Samson LD (2002). Damage recovery pathways in Saccharomyces cerevisiae revealed by genomic phenotyping and interactome mapping.. Mol Cancer Res.

[15] Jelinsky SA, Samson LD (1999). Global response of Saccharomyces cerevisiae to an alkylating agent.. Proc Natl Acad Sci U S A.

[16] Lee MW, Kim BJ, Choi HK, Ryu MJ, Kim SB (2007). Global protein expression profiling of budding yeast in response to DNA damage.. Yeast.

[17] Murray JI, Whitfield ML, Trinklein ND, Myers RM, Brown PO (2004). Diverse and specific gene expression responses to stresses in cultured human cells.. Mol Biol Cell.

[18] Wiles AM, Ravi D, Bhavani S, Bishop AJ (2008). An analysis of normalization methods for Drosophila RNAi genomic screens and development of a robust validation scheme.. J Biomol Screen.

[19] Burgis NE, Samson LD (2007). The protein degradation response of Saccharomyces cerevisiae to classical DNA-damaging agents.. Chem Res Toxicol.

[20] Shen C, Lancaster CS, Shi B, Guo H, Thimmaiah P (2007). TOR signaling is a determinant of cell survival in response to DNA damage.. Mol Cell Biol.

[21] Rusyn I, Fry RC, Begley TJ, Klapacz J, Svensson JP (2007). Transcriptional networks in S. cerevisiae linked to an accumulation of base excision repair intermediates.. PLoS ONE.

[22] Dickinson DA, Levonen AL, Moellering DR, Arnold EK, Zhang H (2004). Human glutamate cysteine ligase gene regulation through the electrophile response element.. Free Radic Biol Med.

[23] Dann SG, Thomas G (2006). The amino acid sensitive TOR pathway from yeast to mammals.. FEBS Lett.

[24] Ou YH, Chung PH, Sun TP, Shieh SY (2005). p53 C-terminal phosphorylation by CHK1 and CHK2 participates in the regulation of DNA-damage-induced C-terminal acetylation.. Mol Biol Cell.

[25] Sinnberg T, Lasithiotakis K, Niessner H, Schittek B, Flaherty KT (2008). Inhibition of PI3K-AKT-mTOR Signaling Sensitizes Melanoma Cells to Cisplatin and Temozolomide.. J Invest Dermatol.

[26] Kao HY, Ordentlich P, Koyano-Nakagawa N, Tang Z, Downes M (1998). A histone deacetylase corepressor complex regulates the Notch signal transduction pathway.. Genes Dev.

[27] Kerrien S, Alam-Faruque Y, Aranda B, Bancarz I, Bridge A (2007). IntAct–open source resource for molecular interaction data.. Nucleic Acids Res.

[28] Salwinski L, Miller CS, Smith AJ, Pettit FK, Bowie JU (2004). The Database of Interacting Proteins: 2004 update.. Nucleic Acids Res.

[29] Alfarano C, Andrade CE, Anthony K, Bahroos N, Bajec M (2005). The Biomolecular Interaction Network Database and related tools 2005 update.. Nucleic Acids Res.

[30] Watts DJ, Strogatz SH (1998). Collective dynamics of ‘small-world’ networks.. Nature.

[31] Begley TJ, Samson LD (2004). Network responses to DNA damaging agents.. DNA Repair (Amst).

[32] Lynn S, Yew FH, Hwang JW, Tseng MJ, Jan KY (1994). Glutathione can rescue the inhibitory effects of nickel on DNA ligation and repair synthesis.. Carcinogenesis.

[33] Mizumoto K, Glascott PA, Farber JL (1993). Roles for oxidative stress and poly(ADP-ribosyl)ation in the killing of cultured hepatocytes by methyl methanesulfonate.. Biochem Pharmacol.

[34] Harper JW, Elledge SJ (2007). The DNA damage response: ten years after.. Mol Cell.

[35] Baudot A, Angelelli JB, Guenoche A, Jacq B, Brun C (2008). Defining a modular signalling network from the fly interactome.. BMC Syst Biol.

[36] Barrios-Rodiles M, Brown KR, Ozdamar B, Bose R, Liu Z (2005). High-throughput mapping of a dynamic signaling network in mammalian cells.. Science.

[37] Hwang D, Smith JJ, Leslie DM, Weston AD, Rust AG (2005). A data integration methodology for systems biology: experimental verification.. Proc Natl Acad Sci U S A.

[38] Vidal M (2005). Interactome modeling.. FEBS Lett.

[39] Jones S, Zhang X, Parsons DW, Lin JC, Leary RJ (2008). Core signaling pathways in human pancreatic cancers revealed by global genomic analyses.. Science.

[40] Lin J, Gan CM, Zhang X, Jones S, Sjoblom T (2007). A multidimensional analysis of genes mutated in breast and colorectal cancers.. Genome Res.

[41] Ghobrial IM, Witzig TE, Adjei AA (2005). Targeting apoptosis pathways in cancer therapy.. CA Cancer J Clin.

[42] Rizzo P, Osipo C, Foreman K, Golde T, Osborne B (2008). Rational targeting of Notch signaling in cancer.. Oncogene.

[43] McConnell JL, Gomez RJ, McCorvey LR, Law BK, Wadzinski BE (2007). Identification of a PP2A-interacting protein that functions as a negative regulator of phosphatase activity in the ATM/ATR signaling pathway.. Oncogene.

[44] Chou WC, Wang HC, Wong FH, Ding SL, Wu PE (2008). Chk2-dependent phosphorylation of XRCC1 in the DNA damage response promotes base excision repair.. Embo J.

[45] Zhao Y, Katzman RB, Delmolino LM, Bhat I, Zhang Y (2007). The notch regulator MAML1 interacts with p53 and functions as a coactivator.. J Biol Chem.

[46] Langie SA, Knaapen AM, Houben JM, van Kempen FC, de Hoon JP (2007). The role of glutathione in the regulation of nucleotide excision repair during oxidative stress.. Toxicol Lett.

[47] Jelinsky SA, Estep P, Church GM, Samson LD (2000). Regulatory networks revealed by transcriptional profiling of damaged Saccharomyces cerevisiae cells: Rpn4 links base excision repair with proteasomes.. Mol Cell Biol.

[48] Barlow C, Hirotsune S, Paylor R, Liyanage M, Eckhaus M (1996). Atm-deficient mice: a paradigm of ataxia telangiectasia.. Cell.

[49] Boutros M, Kiger AA, Armknecht S, Kerr K, Hild M (2004). Genome-wide RNAi analysis of growth and viability in Drosophila cells.. Science.

[50] Livak KJ, Schmittgen TD (2001). Analysis of relative gene expression data using real-time quantitative PCR and the 2(−Delta Delta C(T)) Method.. Methods.

[51] Costa LdF, Rodrigues FA, Travieso G, Boas PRV (2007). Characterization of complex networks: A survey of measurements.. Advances in Physics.

[52] Sokal RR, Rohlf FJ (1969). Biometrics.

[53] Fisher R (1922). On the interpretation of χ2 from contingency tables, and the calculation of P.. Journal of the Royal Statistical Society.

