# Correction: A Network of Conserved Damage Survival Pathways Revealed by a Genomic RNAi Screen

**DOI:** 10.1371/annotation/526db6e9-0ba5-4ec6-a257-2befb76f34b7

**Published:** 2009-10-19

**Authors:** Dashnamoorthy Ravi, Amy M. Wiles, Selvaraj Bhavani, Jianhua Ruan, Philip Leder, Alexander J. R. Bishop

Table S7 is incorrect. The correct version can be viewed here: 
