# Supplementary material for: A Network of Conserved Damage Survival Pathways Revealed by a Genomic RNAi Screen
Source: PLoS Genet. 2009 Jun 19;5(6):e1000527. doi: 10.1371/journal.pgen.1000527 (PMC2688755; doi:10.1371/journal.pgen.1000527)
Supplement: Table S5 — Summary of the number of MMS survival genes identified per MMS survival pathway (BER: base excision repair; DDR: DNA damage response; Proteasome; GSH: glutathione synthesis; TOR: TOR pathway; NER: nucleotide excision repair; MMR: mismatch repair; HRR: homologous recombination repair; RECQ: RecQ helicases; Transcription: basal transcription; Ribosome; ATPase; Notch: Notch signaling pathway), and the percentage non-essential genes of each pathway that this represents. Protein enrichment within each pathway compared to the number of genes that validated (202 of 13826 prior to pathway analysis and 307 of 13826 including pathway analysis) is determined using a Fisher's Exact Test (NS: Not Significant; NA: Not Applicable). (0.51 MB PDF) [file pgen.1000527.s015.pdf]

MMS Survival Pathways

			Screen Hits						Not Screen Hits								
	Genes in Pathway	Essential Genes in Pathway	Validated	P= (Validated)	Trend	P= (Validated + Trend)	Did not validate	Adjusted for Essential Genes	Validated	Trend	Total Genes Validated	P= (Total Validated)	P= (Adjusted for Essential Genes)	Total (Validated + Trend)	P= (Total Validated + Trend)	P= (Adjusted for Essential Genes)	Percent of testable Pathway Genes that Validated
BER	20	0	2	3.40E-02	4	3.48E-07	0	NA	6	1	8	2.64E-10	NA	13	2.83E-17	NA	65%
DDR	22	1	1	NS	0	NA	0	NS	6	4	7	2.27E-08	1.57E-08	11	4.44E-13	2.27E-13	52%
Proteasome	51	15	5	9.50E-04	0	NA	0	1.80E-04	15	2	20	2.54E-23	3.20E-27	22	7.36E-23	2.53E-27	61%
GSH	60	0	3	NS	0	NA	0	NA	18	4	21	3.71E-23	NA	25	2.88E-25	NA	42%
TOR	52	0	6	1.20E-04	1	1.20E-05	2	1.10E-04	16	6	22	2.33E-26	1.37E-26	31	2.94E-37	1.21E-37	61%
NER	39	0	9	5.27E-09	1	2.44E-10	1	NA	11	3	20	2.72E-26	NA	25	1.36E-31	NA	64%
MMR	18	0	0	NA	1	NS	0	NA	4	2	4	1.24E-04	NA	7	7.42E-08	NA	39%
HRR	11	0	2	1.10E-02	0	NA	0	NA	2	2	4	1.45E-05	NA	6	5.36E-08	NA	55%
RECQ	4	0	1	NS	0	NA	0	NA	0	0	1	NA	NA	1	NA	NA	25%
Transcription	32	3	6	6.96E-06	1	3.92E-07	5	3.79E-06	6	7	12	2.32E-14	5.58E-15	25	3.58E-35	2.71E-37	86%
Ribosome	64	9	3	NS	0	NA	3	4.80E-02	7	20	10	4.10E-09	8.97E-09	32	5.25E-35	6.64E-38	58%
ATPase	46	5	4	4.70E-03	1	5.88E-04	2	3.10E-03	14	4	18	3.81E-21	2.94E-22	25	5.33E-29	8.90E-31	61%
Notch	20	2	3	3.00E-03	0	NA	0	2.20E-03	5	4	8	2.64E-10	9.43E-11	12	1.95E-15	2.99E-16	67%
