# Supplementary figures and images for: A Network of Conserved Damage Survival Pathways Revealed by a Genomic RNAi Screen
Source: PLoS Genet. 2009 Jun 19;5(6):e1000527. doi: 10.1371/journal.pgen.1000527 (PMC2688755; doi:10.1371/journal.pgen.1000527)

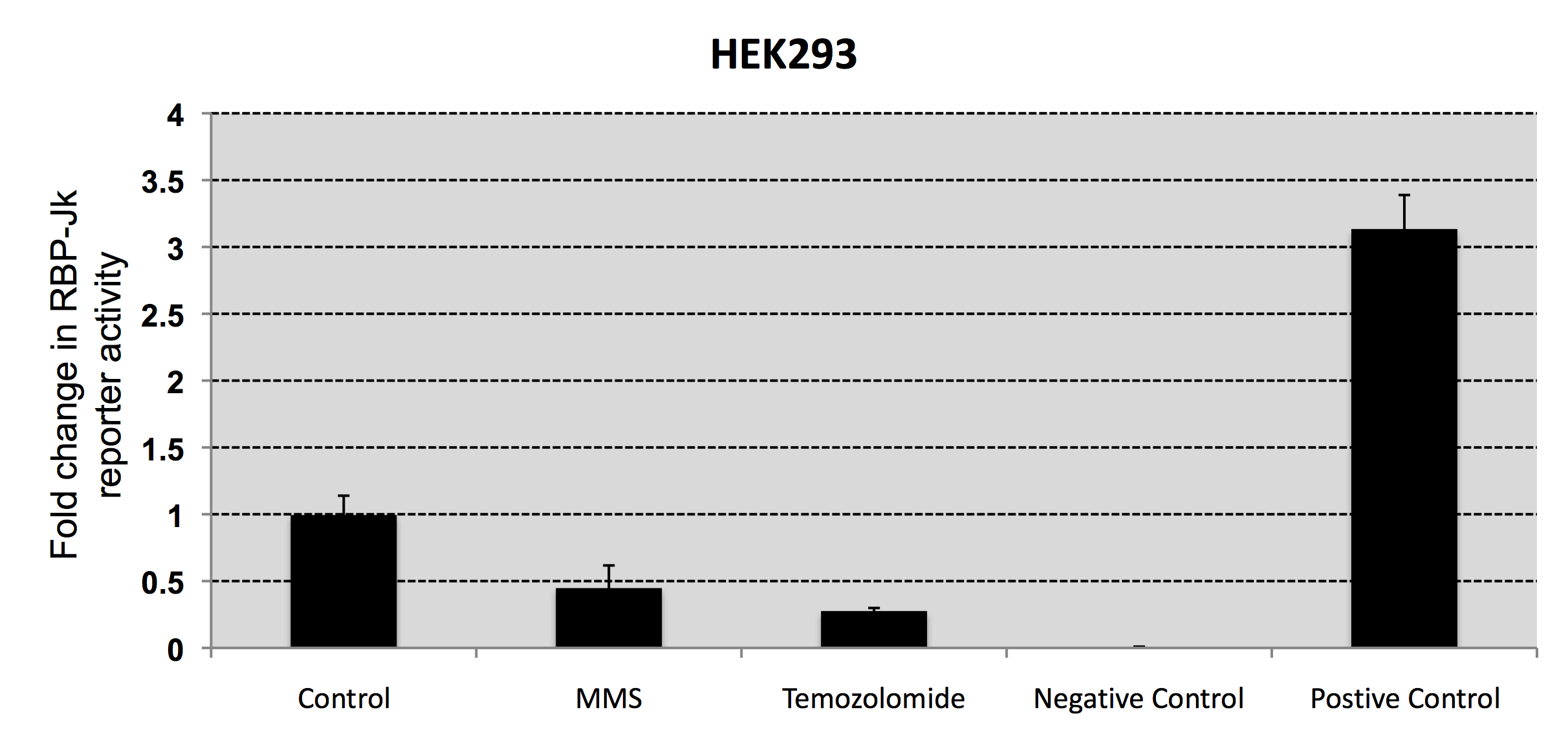

Supplement: Table S7 — Connectivity analysis of MMS survival genes identified by the RNAi genomic screen and following validation analyses. Provided are the number of proteins identified, the number of those proteins that are within the protein:protein interactome (PPI), the number of direct connections between these proteins, the average distance between every possible pair of proteins within the network compared to the expected values, the global efficiency and the clustering coefficient. For each measurement the expected number of direct interactions is derived from the same number of proteins randomly selected 1000 times from the PPI. Analyses are provided for the proteins identified by the MMS screen prior to validation (Screen hits), those screen hits that actually validated (Validated hits), the validated hits as well as essential proteins that are connected to two or more hits (Validated+essential connectors), the total number of validated MMS survival proteins identified by both screen and pathway validation (Validated+pathway hits), the Validated and pathway hits and the essential proteins that are connected to two or more hits (Validated+pathway hits+essential connectors), the total number of validated MMS survival proteins and all other components of the 13 MMS survival pathways (Validated+all in pathways), and finally the total number of validated MMS survival proteins, all other components of the 13 MMS survival pathways and the essential proteins that are connected to two or more hits or pathway proteins (Validated+all in pathways+essential connectors). Analyses were also provided to compare connectivity of real and randomly rewired PPI. (0.19 MB DOC) [file pgen.1000527.s017.doc]
