# Supplementary material for: A Network of Conserved Damage Survival Pathways Revealed by a Genomic RNAi Screen
Source: PLoS Genet. 2009 Jun 19;5(6):e1000527. doi: 10.1371/journal.pgen.1000527 (PMC2688755; doi:10.1371/journal.pgen.1000527)
Supplement: Text S1 — Methods for 8-oxo-guanine assay and notch reporter analysis. (0.03 MB DOC) [file pgen.1000527.s018.doc]

Supplementary Methods

**8-oxo-deoxy Guanine assay.**

To measure the levels of 8-oxo-deoxy guanine, the oxyDNA assay kit-fluorometric (EMD Chemicals, Inc., Gibbstown, NJ) was used following the manufacturer’s instructions. Briefly, following protein knock-down, cells were incubated for additional 24 h with or without exposure to MMS in a 384-well plate, fixed using 4% paraformaldehyde, dehydrated using graded methanol and permeabilized using 99% methanol. Following permeabilization, cells were rehydrated and blocked using blocking solution supplied with the kit. Cells were then incubated with FITC conjugated antibody overnight at 4 ºC, followed by three washes with wash buffer supplied. Images of cells were acquired using a Carl Zeiss Axiovert 200M inverted microscope using 40X objective and 488 nm filter set. The percent of cells positive for fluorescence was determined by counting total cells and labelled cells in each field. Assays were performed in quadruplicate.

**Notch reporter assay.**

To analyze the function of the Notch pathway, we measured the transcriptional activity of its downstream component RBP-Jκ, using a Cignal reporter assay kit (SABiosciences, Fredrick, MD). The assay kit consists of a DNA construct to monitor luciferase activity of the RBP-Jκ reporter and renilla luciferase to monitor transfection efficiency. HEK 293 cells were transfected with reporter or control plasmids provided in the kit in 96 well plate, using SureFECT transfection reagent (SABiosciences, Fredrick, MD), following the manufacturer’s protocol. Transfected cells were incubated for 48 hours, exposed to damaging agent, and incubated for additional 24 hours. Luciferase activity was quantified and normalized with Renilla luciferase for transfection efficiency, using Dual-Glo luciferase assay kit (Promega). Assays were performed in quadruplicate.
